# A human endogenous retrovirus-derived gene that can contribute to oncogenesis by activating the ERK pathway and inducing migration and invasion

**DOI:** 10.1371/journal.ppat.1006451

**Published:** 2017-06-26

**Authors:** Cécile Lemaître, Jhen Tsang, Caroline Bireau, Thierry Heidmann, Marie Dewannieux

**Affiliations:** 1 CNRS, UMR 9196, Institut Gustave Roussy, Villejuif, France; 2 Université Paris-Sud, Orsay, France; 3 Université Paris Denis Diderot, Sorbonne Paris-Cité, Paris, France; University of Illinois at Chicago College of Medicine, UNITED STATES

## Abstract

Endogenous retroviruses are cellular genes of retroviral origin captured by their host during the course of evolution and represent around 8% of the human genome. Although most are defective and transcriptionally silenced, some are still able to generate retroviral-like particles and proteins. Among these, the HERV-K(HML2) family is remarkable since its members have amplified relatively recently and many of them still have full length coding genes. Furthermore, they are induced in cancers, especially in melanoma, breast cancer and germ cell tumours, where viral particles, as well as the envelope protein (Env), can be detected. Here we show that HERV-K(HML2) Env *per se* has oncogenic properties. Its expression in a non-tumourigenic human breast epithelial cell line induces epithelial to mesenchymal transition (EMT), often associated with tumour aggressiveness and metastasis. In our model, this is typified by key modifications in a set of molecular markers, changes in cell morphology and enhanced cell motility. Remarkably, microarrays performed in 293T cells reveal that HERV-K(HML2) Env is a strong inducer of several transcription factors, namely ETV4, ETV5 and EGR1, which are downstream effectors of the MAPK ERK1/2 and are associated with cellular transformation. We demonstrate that HERV-K(HML2) Env effectively activates the ERK1/2 pathway in our experimental setting and that this activation depends on the Env cytoplasmic tail. In addition, this phenomenon is very specific, being absent with every other retroviral Env tested, except for Jaagsiekte Sheep Retrovirus (JSRV) Env, which is already known to have transforming properties *in vivo*. Though HERV-K Env is not directly transforming by itself, the newly discovered properties of this protein may contribute to oncogenesis.

## Author summary

Nearly half the DNA of mammals consists of reitarated, selfish elements that can move and amplify within the genome. With time, some of these elements are recruited by the host and the proteins they encode are used to fulfill physiological functions, whereas other elements have conserved some of their pathological properties and contribute to the development of diseases. The human HERV-K(HML2) elements originated from an ancestral infection of the primate germline by an infectious retrovirus that has been maintained and amplified in the human lineage. It is associated with several pathologies in modern humans, in particular cancer of the breast, germline and skin. We show that the HERV-K(HML2) envelope protein is able to activate a major cellular signalling pathway often involved in human cancers, and that its expression promotes a series of cellular changes that are characteristic of cancer development. Altogether, this study indicates that the expression of HERV-K(HML2) elements is not only a marker of cancer, but can also directly participate to tumourigenesis via the newly discovered oncogenic properties carried by the envelope protein.

## Introduction

Retroviruses are responsible for a broad range of diseases in animals and humans, the most common of which is the development of cancers. The mechanisms by which they contribute to oncogenesis are diverse and include: (i) insertional mutagenesis, due to activation of cellular proto-oncogenes by inserted proviruses, (ii) immunosuppression, by an immunosuppressive domain conserved in most retroviral envelope proteins and (iii) direct oncogenic activity, with some retroviruses encoding proteins with transforming activities leading to tumour formation. For example JSRV causes the development of contagious lung tumours in sheep [1], and the Env protein alone has been shown to be responsible for the formation of the tumours in vivo [2]. It is also able to transform cell lines [3–8] and induce lung tumour formation in mice [9]. The transforming pathways involved are many, and depend on the direct action of Env itself, as well as the Env-receptor interaction [1].

Endogenous retroviruses (ERVs) are the remnants of past retroviral infections, which have been captured by the host during the course of evolution. They occupy around 8% of the human genome and are similar to the proviral forms of integrated retroviruses from which they derive. Whilst most ERVs are defective and have degenerated over time, others have retained some or all of their open reading frames (ORFs) and can encode potentially pathogenic viral proteins [10–13]. These elements are normally suppressed in healthy tissues but expression has been reported in animal and human cancers [14–17]. The HERV-K (HML2) family (hereafter shortened to HERV-K) is remarkable in that it has recently amplified in humans and many of its ORFs are intact, making it the largest contributor of retroviral-derived proteins in the genome [18]. Expression of the associated proteins and viral particles has been detected in cell lines as well as in human cancers, including melanoma, breast and ovarian cancers [19–22]. In addition, reports indicate that HERV-K expression is important for the transformed phenotype of several cell lines. For example, in melanoma, downregulation of HERV-K Env by siRNA decreases the tumorigenic potential of the A-375 cell line [23] and HERV-K Env expression in the TVMA-12 cell line is necessary for the transition from a adherent to a non-adherent phenotype [24]. In several breast cancer-derived cell lines, HERV-K expression was also recently shown to be important for cell motility and growth, both in vitro and in vivo [25].

In this study, we investigated whether HERV-K Env could have oncogenic properties and be involved in the transformation process of the cells where it is expressed. We demonstrate that its expression induces epithelial to mesenchymal transition (EMT), leading to an increase in cell motility. We also show that HERV-K Env activates the ERK1/2 pathway and promotes the expression of transcription factors involved in oncogenesis.

## Results

### HERV-K Env induces EMT in the MCF10A breast cell line

**Fig 1:**
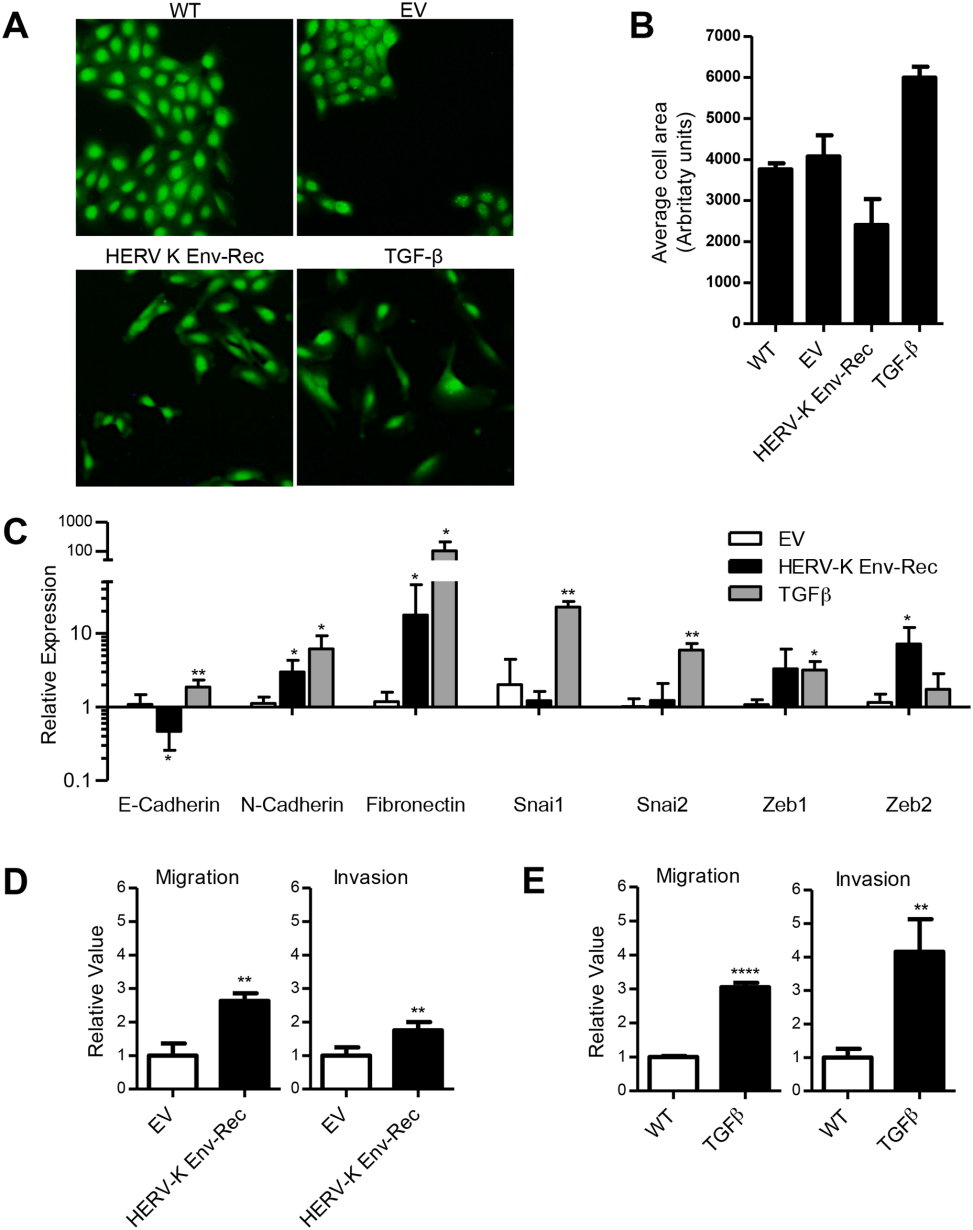
HERV-K envelope protein induces EMT in MCF10A human breast epithelial cell line. MCF10A cells were transduced with either a control “empty” vector (EV) or a vector expressing HERV-K Env-Rec. (**A**) Fluorescence microscopy images of MCF10A cell populations after selection with hygromycin compared to control wildtype cells (WT) and cells stimulated with 5ng/ml TGFβ (TGFβ). Images were taken at 10x magnification after staining the cells with Cell Tracker Green. (**B**) Changes in cell morphology were quantified by calculating the average cell size (area) of each population (a minimum of 1000 cells were counted for each population). (**C**) The expression levels of EMT related transcripts were evaluated in the different MCF10A populations by qRT-PCR. Genes of interest were normalized to the RPLO gene and expressed relative to WT cells. (**D**+**E**) The migration and invasion capacities of the cells were assessed using transwell assays. The cells were allowed to migrate and invade (through a layer of Matrigel) for 22 hours. DAPI staining was performed and cells that had migrated/invaded were observed under a fluorescent microscope and counted. Migration and invasion of TGF stimulated cells and HERV-K Env expressing cells is represented relative to WT and EV cells respectively. Error bars represent the standard deviation of at least 3 independently generated cell populations with triplicate readings taken from each population. Significant differences between cell populations were assessed using a one-tailed t test performed relative to WT cells (C+E) and EV cells (D); * = p<0.05; ** = p<0.01; *** = p<0.001.

As HERV-K Env expression has been reported in several human cancers, including breast, we investigated whether it could have a causal role in the transformation process. For this, we used the non-transformed breast epithelial MCF10A cell line, widely used to study the process of oncogenesis [26, 27]. Stable long-term expression of the HERV-K Env was obtained following lentiviral transduction. A control population was generated using an empty vector. After selection, transgene expression in the populations was measured by qRT-PCR using primers located in a region common to both vectors, and were found to express similar levels of lentiviral transcripts. The HERV-K Env population alone was also found to express HERV-K Env transcripts at high but still physiological level (S1 Fig), and as expected, we also detected expression of Rec transcripts that are produced from the Env construct through internal splicing [28, 29]. Interestingly, the HERV-K Env populations displayed an altered morphology (Fig 1A), changing size (Fig 1B) and becoming more elongated. They also lost their typical acinus organisation, and are dispersed in the plate, a phenotype reminiscent of that observed on cells treated with TGFß, a known inducer of EMT. EMT is a process during which cells change their identity, and is important both in normal development and in epithelial cancer progression. It is characterized phenotypically by a loss of cell polarity and cell-cell adhesion, and at the molecular level by a decrease of E-cadherin, an increase of N-cadherin and fibronectin, as well as the induction of a few key transcription factors (mainly Snai1, Snai2, Zeb1, Zeb2) [30]. We quantified these markers in the different populations. As expected, TGFß-treated cells showed an increase of the expression of the mesenchymal markers fibronectin and N-cadherin, as well as EMT-associated transcription factors Snai1&2 and to a lesser extent Zeb1 (Fig 1C). Interestingly, HERV-K Env, but not the control, also modified the expression of EMT-associated genes, with a significant increase in the levels of fibronectin and N-cadherin, and a decrease in E-cadherin. However, the most induced transcription factor was Zeb2, unlike in TGFß treated cells. We also tested the different populations for changes in their motility properties using transwell assays. As shown in Fig 1D, the HERV-K Env expressing populations displayed an increase in cell migration and invasion, similar to what is observed with TGFß-treated cells (Fig 1E). Altogether the data obtained with the MCF10A cells indicate that HERV-K Env possesses oncogenic properties, and modifies the cell physiology to induce a process related to, but not identical to TGFß-induced EMT.

### HERV-K Env induces the expression of transcription factors associated with oncogenesis

**Fig 2:**
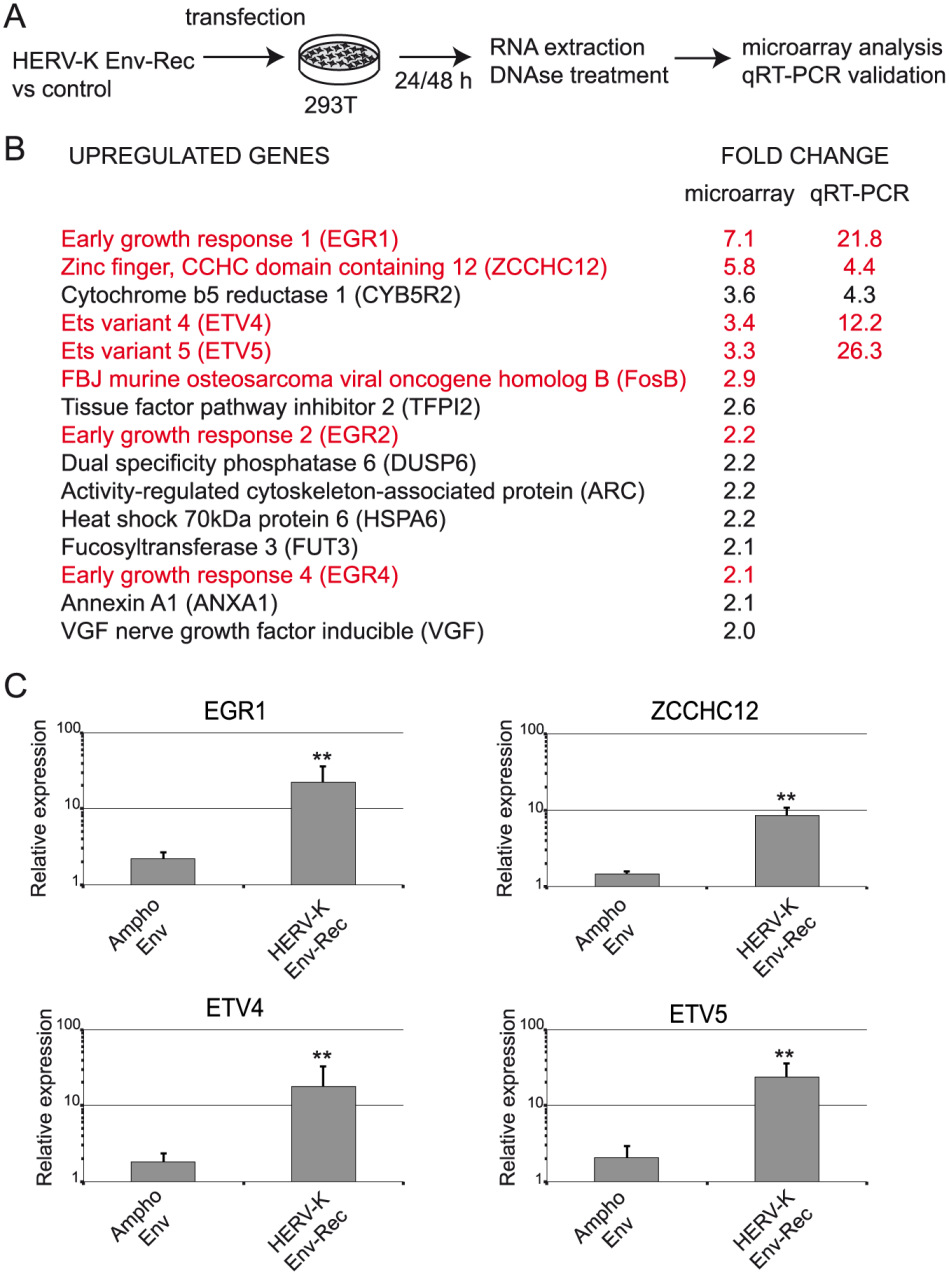
HERV-K envelope protein induces expression of several transcription factors. **(A)** Scheme of the experimental procedure. 293T cells were transfected with the indicated plasmids, in duplicate for each condition. 24 and 48 hours post transfection, cells were harvested for RNA extraction. Gene expression analysis was performed with the Agilent SurePrint G3 Human GE 8x60K Microarray. **(B)** Data from microarrays were processed and normalized as described in the material and methods in order to assess differentially expressed genes between control and HERV-K Env expressing samples. The genes listed here were from the 48 hour time point and selected by the following criteria: fold-change ≥2 and a false detection rate (FDR) <0.05. Expression levels of the top five genes were confirmed by qRT-PCR performed on the same samples. The results after normalisation with RPLO gene are indicated in the right column. **(C)** qRT-PCR was performed on total RNA extracted from Ampho Env (control) or HERV-K Env-Rec expressing cells at 48 hours post-transfection. The amount of mRNA transcripts for the indicated transcription factors (EGR1: Early Growth Response 1; ETV4 and ETV5: Ets variant 4/5) were normalised to the RPLO gene and expressed relative to non-transfected 293T cells. Error bars represent the standard deviation of five independent experiments. Significant differences in gene expression were assessed using a one-tailed t test performed relative to the Ampho Env condition.

To further characterise HERV-K Env effects on cell physiology, we used the embryonic kidney 293T cell line. We searched for genes whose expression is modified by HERV-K Env using a non-biased transcriptomic approach. 293T cells were transfected in duplicates with a vector expressing the HERV-K Env or a control plasmid, under conditions adjusted to minimise cell toxicity due to the transfection (Fig 2A). Of note, the two expression vectors are identical except for nt 6759 and 6762 that introduce premature stop codons in the control vector, leading to the production of a much shorter protein (102 aa instead of 699), in order to rule out any effect due to the Env RNA per se. The transcriptomes were compared at 24 and 48 hours post transfection using whole-genome microarrays. Genes with statistically significant changes in expression (≥2-fold) were further analysed. At 24 hours post-transfection, no significant difference between the two conditions was observed, probably due to very low protein expression levels at this time point. At 48 hours, we found 86 genes with modified expression levels (69 down-regulated, 17 up-regulated). After checking, the down-regulated genes proved to be genes induced by the transfection itself. The expression level of these genes increased between 24h and 48h after transfection, but to a lower extent in the HERV-K Env cells than in the control, leading to a seemingly downregulation of these genes by HERV_K Env when only the 48h expression data are taken into account. These genes were not investigated further.

Amongst the identified upregulated genes (Fig 2B), there was a strong excess of transcription factors (7 out of 17, with five in the top six most upregulated genes). Interestingly, they include EGR1, ETV4, ETV5 and FosB that have been associated with EMT and tumour aggressiveness in several cancers [31–35]. We confirmed the induction of the top five genes from the list in the same samples by qRT-PCR (Fig 2B). We noted some variations in the measurements between microarray and qRT-PCR data, but this is likely due to the specific sequences of the primers/probes used in each technique. We also verified that the induction of transcription factors was specific and not observed with another retroviral Env protein using an expression vector for the Amphotropic Murine Leukemia Virus Env as a control in an independent series of experiments. As shown in Fig 2C, qRT-PCR confirmed the increased expression of the four transcription factors (EGR1, ZCCHC12, ETV4 and ETV5) by HERV-K Env.

### HERV-K Env activates ERK1/2, like the oncogenic JSRV Env

**Fig 3:**
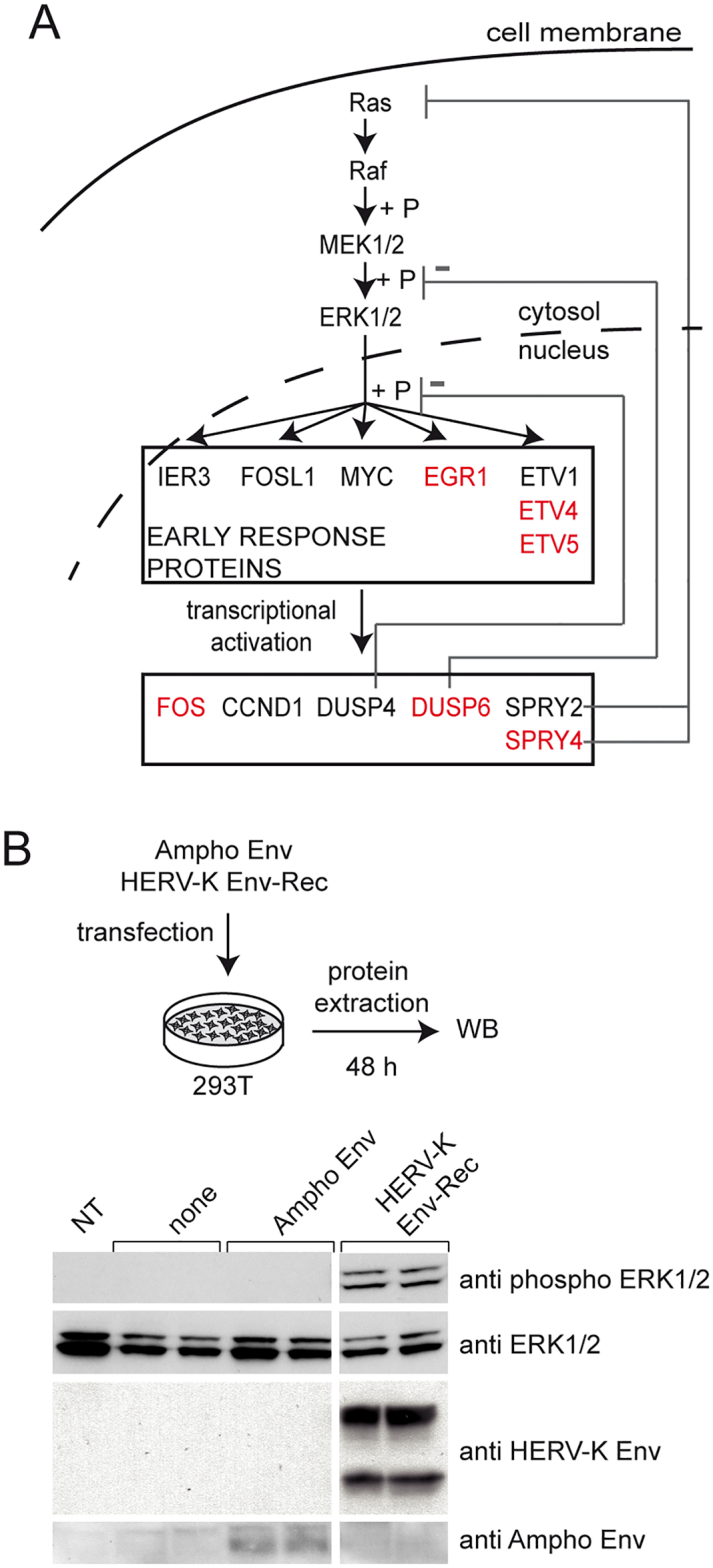
HERV-K Env activates the ERK1/2 MAPK. **(A)** Overview of the ERK1/2 MAPK pathway (adapted from [46]). A broad range of extracellular stimuli can activate the ERK1/2 pathway which starts with the activation of Ras. Activated Ras in turn activates Raf, the first kinase in the pathway. Sequential phosphorylation (shown by a +P) and activation of downstream kinases MEK1/2 subsequently activates ERK1/2 which can then translocate to the cell nucleus where it phosphorylates early response targets (including EGR1, ETV4 and ETV5). These targets act as transcriptional activators of several genes. Genes identified in the microarray data presented in Fig 2B are highlighted in red. **(B)** Activation of ERK1/2 was directly tested in 293T cells transfected with HERV-K Env-Rec (or indicated control proteins). Western Blots were performed on cell lysates harvested 48 hours post transfection and membranes were probed for phosphorylated ERK1/2, stripped and stained again for the total form of the kinases. (NT: non transfected). Expression of both HERV-K and Ampho Envs was also confirmed.

We noticed that the majority of genes activated by HERV-K Env are involved in the ERK1/2 MAPK pathway (Fig 3A). Indeed, EGR1, ETV4 and ETV5 are direct targets of ERK1/2. DUSP6, which we found induced at a lower level, is a secondary target involved in the negative retro-control of the pathway. This strongly suggested that HERV-K Env is an inducer of the ERK1/2 pathway. We tested this hypothesis by assessing the phosphorylation of ERK1/2 following transient transfection of 293T cells (Fig 3B). As shown, cells transfected with HERV-K Env show a marked phosphorylation of ERK1/2, not seen with the controls, while total amounts of ERK1/2 are similar with all plasmids.

**Fig 4:**
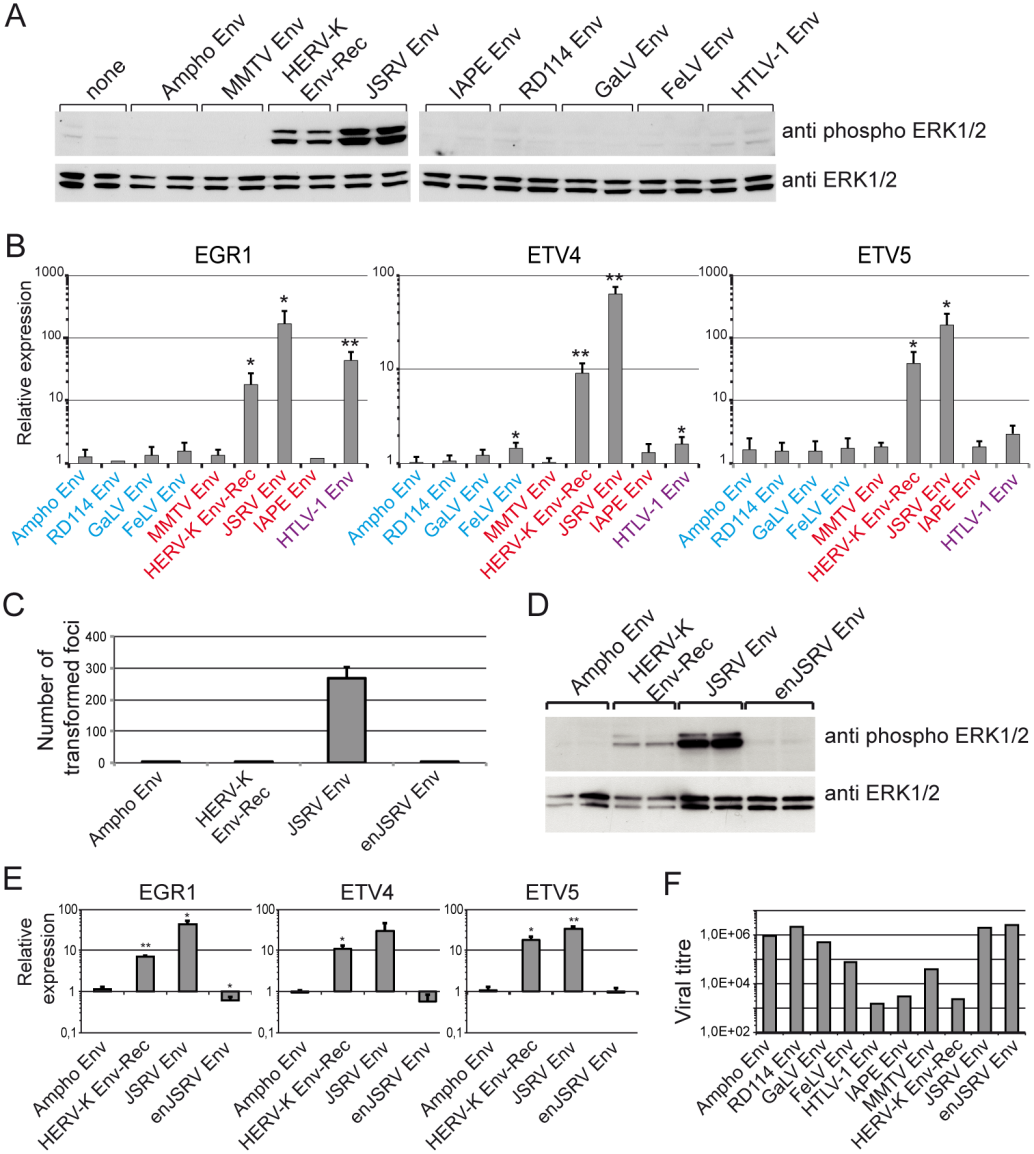
Oncogenic properties carried by different retroviral Envs. **(A)** Phosphorylation of ERK1/2 in 293T cells expressing a panel of retroviral Envs was assayed as described in Fig 3B. Samples in left and right panels were prepared and processed (migration, transfer, revelation) at the same time; therefore intensity of the signals in both sets of panels can be compared directly. The Envs tested span three different retroviral genera and are colour-coded according to group (red: betaretroviruses; blue: gammaretrovirus; purple: deltaretrovirus). **(B)** The mRNA levels of the transcription factors EGR1, ETV4 and ETV5 following expression of the indicated Envs were measured as described in Fig 2C. Error bars represent standard deviation of the mean of three independent experiments. (**C**) The transforming activity of Ampho, HERV-K, JSRV and enJSRV retroviral Env proteins was assessed in 208F cells. Briefly, cells were transiently transfected with expression vectors for the 4 Env proteins and left to reach confluence. The number of transformed foci was counted after 3–4 weeks. The histogram represents the average number of foci obtained per 2x10^5^ cells in 3 independent experiments. (**D**) Phosphorylation of ERK1/2 in 293T cells expressing the 4 retroviral Envs mentioned above was assessed as in Fig 3B. (**E**) The mRNA levels of transfection factors EGR1, ETV4 and ETV5 following expression of the 4 Envs was tested as described in Fig 2C. (**F**) We ensured that all the expression vectors for the Env proteins were functional by measuring the viral titres obtained with them when pseudotyping heterologous retroviral particles. Ampho, RD114, GaLV, FelV and MMTV Envs were tested using a MLV core, HERV-K, JSRV, enJSRV, IAP and HTLV1 Envs were tested with a HIV core. Viral titres were measured on 293T cells, except for FeLV pseudotypes (feline G355.5 cells) and MMTV pseudotypes (mouse NIH 3T3 cells). Background infection levels (i.e. the titres obtained with no Env expression vector) were less than 100 whatever the core/target cells combination.

To investigate the specificity of this activation, we transfected a panel of retroviral Envs, encompassing several genera, in 293T cells and measured the expression of EGR1, ETV4 and ETV5, as well as the phosphorylation of ERK1/2. As shown in Fig 4A & 4B, no other retroviral Env was able to induce both ERK1/2 phosphorylation and transcription factor expression, except JSRV Env. Interestingly, JSRV Env’s ability to activate ERK1/2 has been described in other cellular models and has been linked to its strong oncogenic effect [36–38]. This similarity suggests that HERV-K Env possesses oncogenic properties as well. Of note, the closely related MMTV Env was unable to activate the ERK1/2 pathway, indicating that the ability to activate the ERK1/2 pathway is not a general property of betaretroviral Envs. Finally, EGR1, but not ETV4 and ETV5, was also induced by the deltaretroviral HTLV-1 Env, consistent with previous data reporting the transactivation of EGR1 promoter by the accessory protein Tax [39] which is also produced from the HTLV-1 Env expression vector. Accordingly, HTLV-1 Env does not induce the phosphorylation of ERK1/2 (Fig 4A).

Due to the similarities with JSRV Env, we tested if expression of HERV-K Env was directly transforming in a classical transformation assay using rat 208F cells. [40, 41] HERV-K Env was unable to induce the formation of transformed foci in this assay, but an otherwise fully infectious functional endogenous allele of JSRV (enJSRV-18) was also negative in this assay (Fig 4C). Furthermore, unlike HERV-K Env, the endogenous JSRV Env was also unable to activate ERK1/2 or induce expression of the transcription factors (Fig 4D+4E). Functional expression of all Env constructs was confirmed by assessing by their ability to produce infectious pseudotyped viral particles (Fig 4E).

### Several present-day alleles of HERV-K Env induce signal transduction

**Fig 5:**
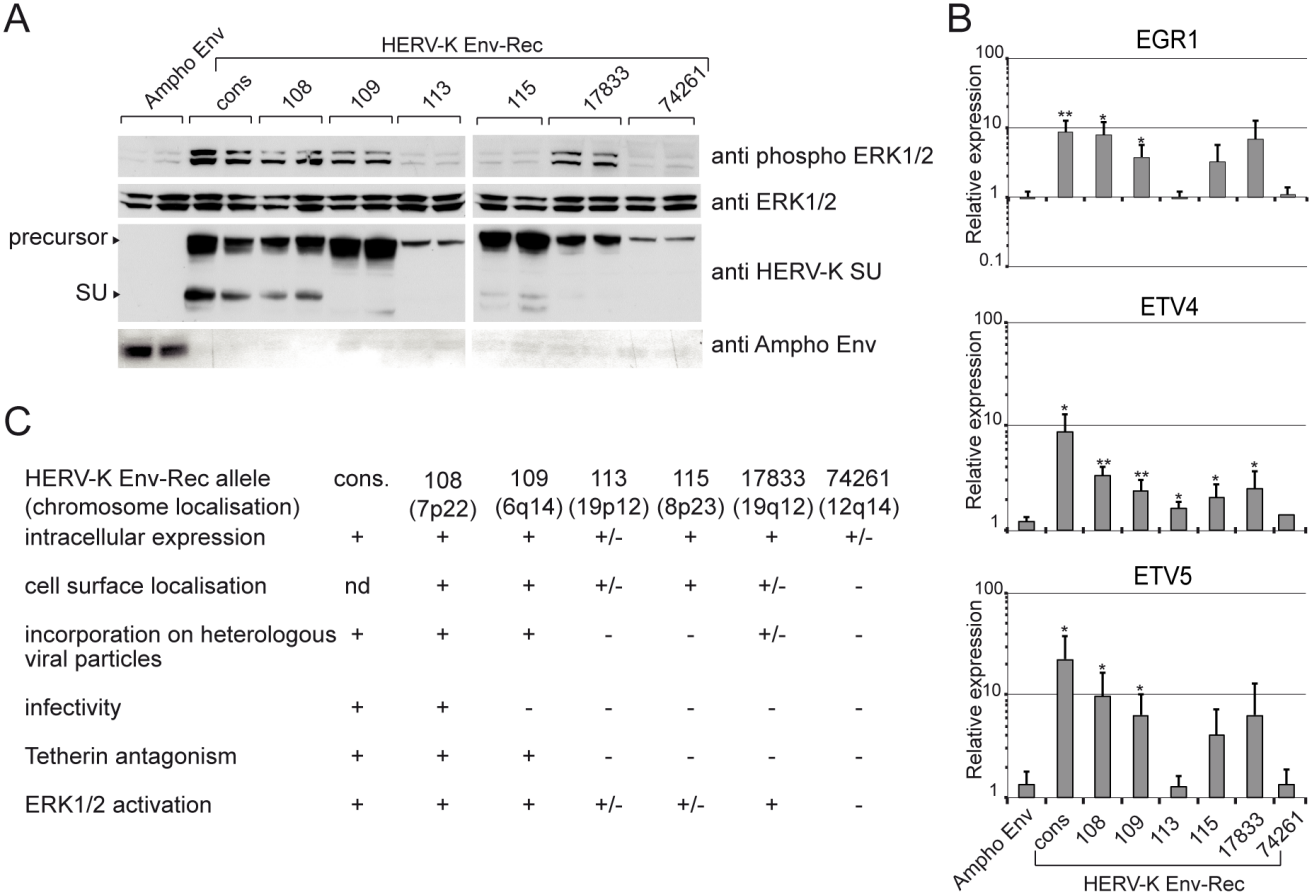
Test for ERK1/2 phosphorylation and expression of transcription factors induced by endogenous HERV-K Env alleles. **(A)** Previously described endogenous alleles of HERV-K Env were tested for their ability to induce phosphorylation of ERK1/2 by Western blot on 293T cell lysates following transient transfection. Expression of each HERV-K Env allele was also assessed. We also checked that Ampho Env was properly expressed. **(B)** Expression levels of the transcription factors EGR1, ETV4 and ETV5 induced by the endogenous alleles of HERV-K Env were measured as described in Fig 2C. Error bars represent the standard deviation of the mean of four independent experiments. (**C**) Recapitulation of the functional properties of all HERV-K Env-Rec variants which have been assessed [43, 61] (nd: not determined).

All the experiments described above have been performed with a consensus HERV-K Env, which theoretically corresponds to the protein in the progenitor virus responsible for the insertion of all modern human-specific HERV-K proviruses [42]. In order to assess the relevance of our observations in modern-day people, we tested the effect of six previously described natural “alleles” of HERV-K Env present in humans on ERK1/2 activation (Fig 5A). Five out of these six, namely K108, K109, K113, K115, K17833 show some transcription factor induction activity (Fig 5B). Among these, the three that correspond to the most functional HERV-K Env proteins when tested for other classical virological properties (Fig 5C) also induce significant ERK1/2 phosphorylation in our assay, suggesting that the ability of the endogenous Envs to activate the signalling pathway is linked to the canonical properties of the infectious retrovirus.

### Identification of the domains in HERV-K Env required to mediate signal transduction

**Fig 6:**
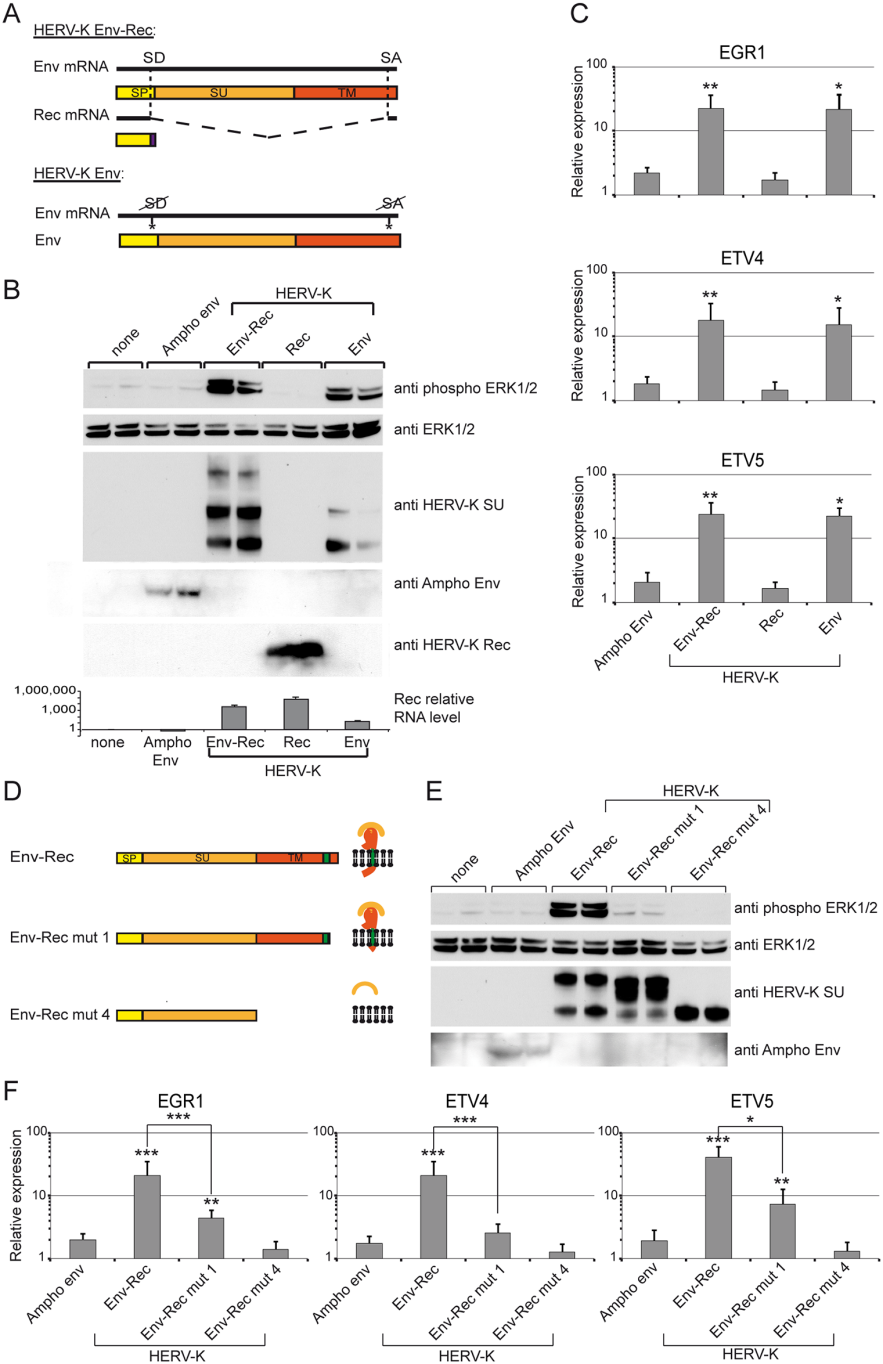
Mapping of the domains involved in HERV-K Env-Rec activation of the ERK1/2 pathway and induction of the transcription factors. (A) The HERV-K Env-Rec plasmid expresses two distinct proteins via alternative splicing: the full-length RNA encodes Env, a glycoprotein that is cleaved into two subunits during synthesis (surface subunit, SU, and transmembrane subunit, TM), while internal splicing sites (SD for splice donor and SA for splice acceptor) lead to the production of the Rec accessory protein. Introducing silent mutations in the splice sites generated an expression vector that expresses Env alone. (B) The different constructs were assayed for their capacity to activate the MAPK ERK1/2 as described in Fig 3B. The expression levels of Ampho Env, HERV-K Env and HERV-K Rec were also checked by Western blot. In the case of Rec, we also performed qRT-PCR reactions on RNA samples to detect specifically the spliced Rec transcripts. (C) Transcription factor expression following transfection of the constructs above was measured as described in Fig 2C. Error bars represent the standard deviation of five independent experiments. (D) Truncation mutants of HERV-K Env were generated to identify the region required for activation of the ERK1/2 pathway. The expected protein structure of each mutant is shown on the right. All modifications were designed to change Env without altering the Rec ORF and keeping the global structure of the Env-Rec RNA. The different domains of each construct are indicated: SP (signal peptide, yellow), SU (orange), TM (red). Env-Rec mut1 corresponds to a C-terminal truncated version of the complete envelope protein. Env-Rec mut4 is the soluble surface subunit. Expression of Ampho Env and the different HERV-K Env-Rec mutants was measured together with their ability to induce phosphorylation of ERK1/2 by Western Blot (E) and expression of the transcription factors was measured by qRT-PCR (F). Error bars represent the standard deviation of the mean of eight independent experiments.

Two proteins are produced from the HERV-K Env-Rec plasmid: the envelope glycoprotein and the accessory protein Rec. The latter consists of two exons and is completely contained within the Env ORF. We wanted to ascertain which of these two proteins mediates the effects seen earlier. We therefore used two previously described vectors [43]: one expressing only Rec, and the other HERV-K Env without Rec (through silent point mutations targeting the splicing sites) (Fig 6A). Expression of the Rec protein did not induce expression of the transcription factors or phosphorylation of ERK1/2, whereas the Env alone was as efficient as the consensus Env-Rec construct to activate signal transduction (Fig 6B & 6C).

We then set about mapping the domains in HERV-K Env required for ERK1/2 activation. Like other retroviral Envs, it is processed by cellular proteases into two subunits, SU, expressed at the cell surface, and TM, containing a single-pass transmembrane domain (Fig 6D). Using previously described mutants [43] (see Fig 6D), we found that the soluble SU (mut 4) completely lost the ability to induce the expression of the transcription factors and the phosphorylation of ERK1/2 (Fig 6E & 6F), indicating that the TM moiety of Env is required. HERV-K Env deleted of its cytoplasmic tail (mut 1) showed some activity, but was markedly decreased. The cytoplasmic tail is therefore important for the activation of the ERK1/2 pathway, either directly or through modification of the intracellular localisation of HERV-K Env, but other domains are also involved.

### HERV-K Env and other cell signalling pathways

**Fig 7:**
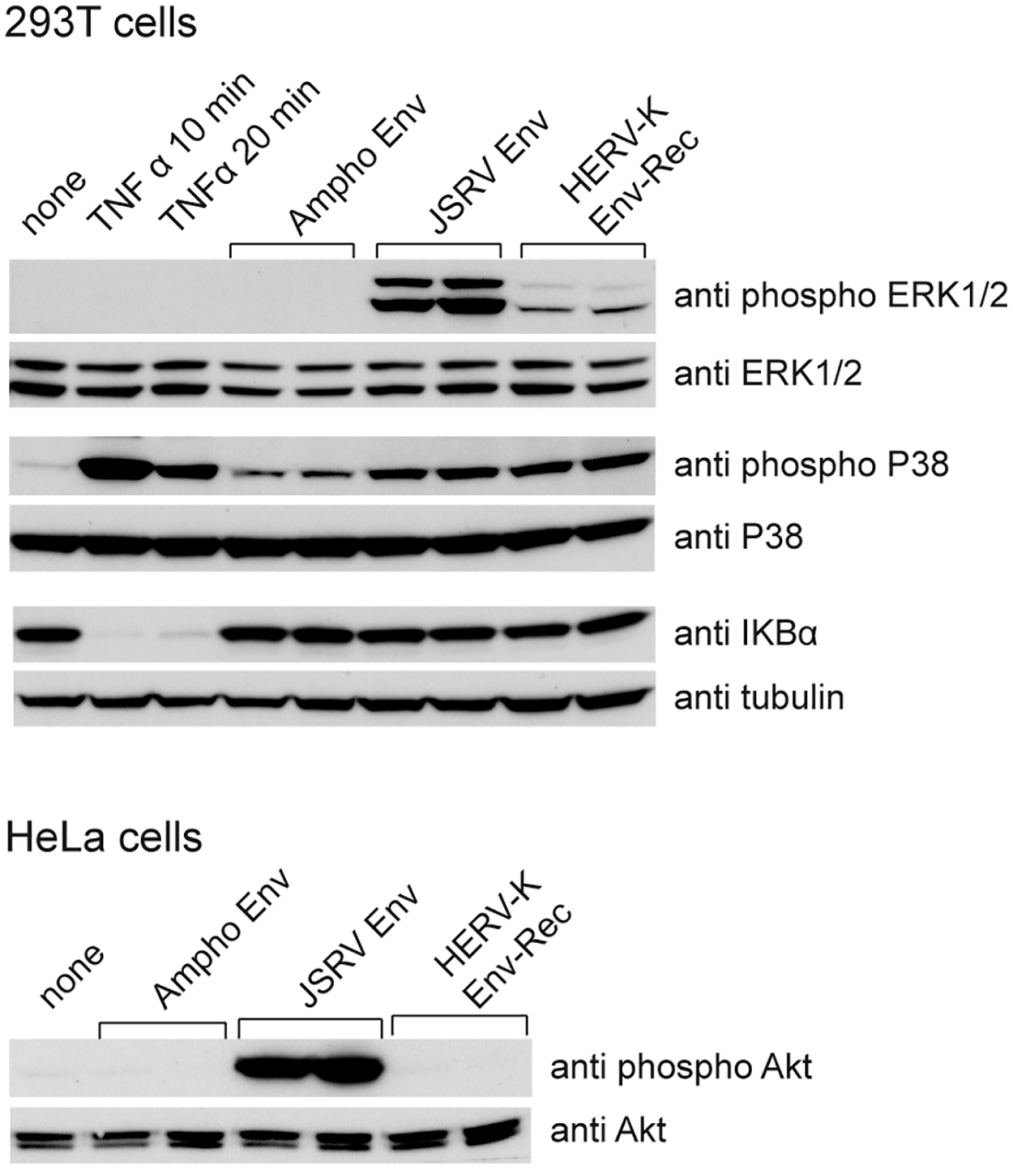
HERV-K Env activation of other signalling pathways. (Top panel) 293T cells were transfected by the indicated plasmids as described in Fig 3B, or stimulated with TNFα (100 ng/μL for 10 and 20 minutes), a positive control for activation of NFκB and p38 pathways. Lysates were tested by Western blot for phosphorylated and total (after membrane stripping) forms of the kinases ERK1/2 and p38, or for IκBα (for IκBα, tubulin was used as a control for homogenous loading). (Bottom panel) HeLa cells were transiently transfected by the indicated plasmids. Cell lysates were harvested 48 hours post transfection and phosphorylated and total (after membrane stripping) levels of AKT were assessed by western blotting.

We also assayed HERV-K Env for potential effects on other cell signalling pathways. We first tested for activation of NFκB, which is often implicated in transformation, using the degradation of IκBα as a sign of activation of the pathway. None of the Envs that we tested had any effect on IκBα levels, unlike TNFα stimulation (Fig 7). HERV-K Env therefore does not modify cell physiology through the NFκB pathway. We then tested for p38 activation (Fig 7). As previously reported, JSRV Env induces the phosphorylation of p38 [36]. The role of p38 activation in JSRV pathogenesis is not clear, but it has been shown to have an inhibitory effect on ERK1/2 signalling. We found that HERV-K Env also activates p38, but our microarray data indicates that ERK1/2 is the major signalling pathway activated by HERV-K Env. It is possible that the activation of p38 by JSRV and HERV-K Envs is a mechanism to regulate the level of ERK1/2 activation. Finally, we tested the PI3K/AKT pathway, previously shown to be important for JSRV Env mediated transformation in several cellular models [6, 8, 40, 41, 44, 45]. Preliminary experiments showed that AKT is constitutively phosphorylated in 293T cells, whatever the culture conditions. We therefore tested for AKT activation in HeLa cells. As shown, HERV-K Env had no effect on the phosphorylation state of AKT, unlike JSRV Env (Fig 7).

### Mechanistic aspects of the activation of the MAP kinase pathway mediated by HERV-K Env

**Fig 8:**
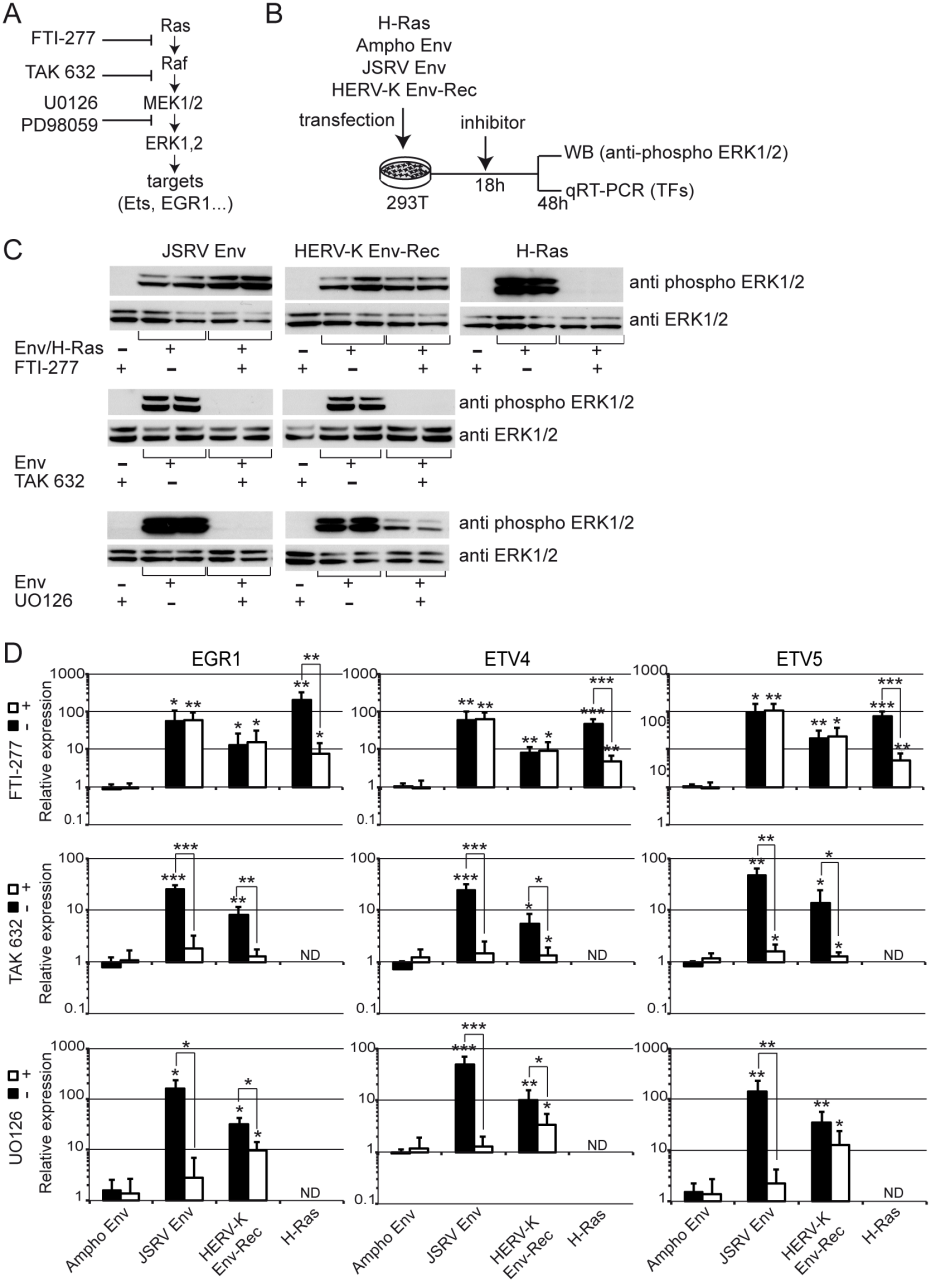
HERV-K Env activates the ERK1/2 MAPK pathway upstream of Raf. **(A)** The ERK1/2 pathway was targeted by different chemical inhibitors: FTI-277 (a H/K-Ras inhibitor), TAK 632 (a pan Raf inhibitor), and U0126 (a broadly-used antagonist of MEK1/2). **(B)** The experimental procedure is illustrated on the scheme. Briefly, 293T cells were transfected with the indicated plasmids. Inhibitors (or vehicle control) were added when media was changed 18 hours post-transfection. Lysates and total RNA were harvested 48 hours post transfection. Activation of ERK1/2 **(C)** and induction of transcription factor expression **(D)** were assayed as described previously (Figs 2C & 3B) in the presence (+) or absence (-) of FTI-277, TAK 632 or U0126. Error bars represent the standard deviation of the mean of five independent experiments.

Finally, using a series of inhibitors (Fig 8A), we characterised where HERV-K Env acts in the ERK1/2 pathway. 293T cells were transfected as before, treated with each inhibitor individually 18h later and ERK1/2 phosphorylation and expression of the transcription factors were measured at 48h (Fig 8B). First, we used FTI-277 that targets H and K-Ras. As expected, this inhibitor efficiently suppressed the phosphorylation of ERK1/2, with a corresponding decrease in transcription factor expression induced by the transfection of a constitutively active H-Ras (Fig 8C & 8D). However, it did not affect the activation of ERK1/2 mediated by HERV-K Env or JSRV Env. This suggests that either these Envs act downstream of Ras or that they activate another form of Ras (e.g. N-Ras). In contrast, TAK632, a potent pan-Raf inhibitor, completely abolished ERK1/2 signal transduction and transcription factor induction by both JSRV and HERV-K Envs, indicating that the two glycoproteins act upstream of the kinase Raf. U0126, a MEK1/2 inhibitor, also impaired the activation of ERK1/2 mediated by HERV-K and JSRV Envs, as expected. Of note, unlike JSRV Env, the inhibition was only partial for HERV-K Env.

## Discussion

In this paper, we report on the pro-oncogenic properties possessed by the Env protein of the HERV-K family and investigate the mechanism of action. Using the MCF10A cell line, we demonstrated that the stable expression of HERV-K Env, at a high but physiologically relevant level (i.e. similar to the expression level naturally observed in some germ cell derived cell lines), induces clear changes in the expression of EMT-associated genes towards a more mesenchymal phenotype, with the cell morphology altered accordingly. Remarkably, these HERV-K Env-induced changes are accompanied by an increase in cell motility. The modification of the attachment proteins we observed is similar to that obtained after treatment with TGFß, but the transcription factors that are induced are different. A number of different transcription factors have been associated with EMT, and this generic term in fact covers several processes that can occur in different circumstances, either in normal development or in the course of disease [30]. Previous studies had already hinted at oncogenic properties for HERV-K Env, but it had only been shown that this Env protein can alter the phenotype of pre-transformed, cancer-derived cell lines [23–25] whereas we demonstrate here that it can also direct non-malignant cells in the path towards transformation. Given the change of the EMT markers and cell motility observed, it is likely that HERV-K Env expression by a tumour or a pre-tumour could also trigger further changes and favour metastasis.

Using microarrays in a different cell model, we identified a very limited number of genes whose expression is induced following HERV-K Env transfection. We showed that the induction of these genes is due to activation of the ERK1/2 pathway in cells following HERV-K Env expression. Most of the identified genes are transcription factors that have already been associated with transformation and cancer. In fact, the list of the induced genes is remarkably similar to that observed in tumours with a mutated BRAF [46]. BRAF-activating mutations have been reported in several tumours, but are particularly common in melanomas [47]. Specific inhibitors of mutated BRAF have been developed and used to treat patients. They promote a dramatic improvement in patient health for up to 6 months until relapse, due to tumours developing resistance to the treatment [48]. HERV-K Env expression in melanomas has been reported by several independent groups [19, 20, 49–52]. It is possible that HERV-K Env expression is part of the mechanism used by the tumours to escape the treatment against mutated BRAF by re-activating the ERK1/2 pathway. Interestingly, a study recently showed that HERV-K expression in several breast cancer cell lines leads to an increase expression of ERK1/2 [25], which indicates that HERV-K expression could increase ERK1/2 signalling by several independent mechanisms.

The consensus HERV-K Env that we used to demonstrate ERK1/2 activation is more efficient than present-day alleles for most canonical virological properties. However, when tested under the same conditions, three out of the six previously characterised full-length “alleles” of HERV-K Env activated ERK1/2 nearly as efficiently as the consensus, giving credence to a possible role of HERV-K proviruses in tumour development. Of interest, these active alleles have been found to be spontaneously expressed in several human cell lines [52, 53]. In addition, the effect of these alleles could be additive, especially when HERV-K proviruses are expressed due to general LTR activation (eg the reported induction by MITF in melanoma [54]), instead of locus-specific expression. Remarkably, no other retroviral Env protein that we tested activated ERK1/2, except for JSRV Env which is known to be a strong oncogene. Furthermore, the oncogenic activity of JSRV Env has been linked to ERK1/2 activation [36, 38], further supporting a role for HERV-K Env in tumour development. The effects observed with JSRV Env are admittedly stronger than those observed with HERV-K Env. However, it should be noted that the JSRV Env used in this study is that encoded by the infectious strain of the virus. Like HERV-K Env, the endogenous JSRV-18 Env was unable to transform 208F cells in our assays and no transforming effect has ever been reported for any of the endogenous copies [1]. It is not surprising that a gene showing such deleterious effects to the host should be lost very quickly following endogenisation. It is quite remarkable that the HERV-K family, which entered the primate lineage more than 40 million years ago, could have conserved some oncogenic properties for so long, even if these properties are slightly subdued. Indeed we showed that a recent, fully infectious endogenous JSRV Env protein has completely lost the ability to activate the ERK1/2 pathway, unlike some HERV-K Env alleles. It is possible that the last amplification of HERV-K elements in the human genomes is the product of a horizontal transmission of an infectious virus that would have remained active in other primate species, instead of the re-activation of previously dormant proviruses. It would be less unexpected for an exogenous, infectious virus to conserve such oncogenic properties that could play an important role in its propagation.

Finally, we used mutant forms of HERV-K Env to map the domains responsible for ERK1/2 activation. We found that the TM subunit of Env is required, and that the cytoplasmic tail plays an important role, although there is still some ERK1/2 activation when it is deleted. Additionally, the 3 endogenous alleles of HERV-K Env-Rec that most strongly activate ERK1/2 all possess an intact cytoplasmic tail (S2 Fig). However, the K115 allele possesses the same sequence and is much less potent for ERK1/2 activation. It is therefore likely that several domains of HERV-K Env cooperate for its oncogenic properties, as demonstrated for JSRV Env for which both SU and TM are involved in the transformation process [55]. Concerning JSRV Env, several intracellular interacting proteins expected to be involved in transformation have been reported recently [56, 57]. In all cases, the reported interacting domain is the Env cytoplasmic tail. Since JSRV and HERV-K Envs show no homology in this region (S3 Fig), these proteins are unlikely to interact with HERV-K Env. Using motif prediction software, we failed to identify any relevant motif in the sequence of the HERV-K Env cytoplasmic tail, and cannot therefore propose a likely mechanism of action. In the future, the elucidation of the cellular proteins interacting with the HERV-K Env cytoplasmic tail could lead to the development of specific inhibitors able to block its oncogenic properties, and could be of therapeutic interest in tumours where HERV-K Env is expressed, providing alternative treatments to those currently available.

## Materials and methods

### Plasmids

HIV-1 derived particles were produced as described [43], using a modified CSGW [58] expressing HERV-K Env or control proteins and the hygromycin resistance gene. pBabe-H-RasG12V [59] was kindly gifted by A. Puisieux. All Env transient expression vectors are CMV-driven. Plasmids for Ampho, IAPE, RD114, GaLV, FeLV and HERV-K Envs have been described previously [60–64]. phCMV-JSRV Env was constructed by replacing the G protein ORF in phCMV-VSV-G (GenBank accession no. AJ318514) with the JSRV Env ORF (plus the 3’ LTR) present in pCMV3JS21ΔGP [3] (a gift from M. Palmarini). The Env gene from the enJSRV-18 provirus was similarly cloned by PCR on sheep genomic DNA using a forward primer on the ATG and the reverse primer indicated in [65]. phCMV-MMTV Env contains the MMTV Env ORF and the 3’ LTR from the pEnv vector [66] (a gift from S. Ross). The HTLV-1 Env plasmid [67] was a gift from C. Pique. phCMV-HERV-K(HML2) Env-LP was derived from phCMV-HERV-K(HML2) Env by changing aa 103 and 104 into stop codons. pCMV-ß (Clontech) is an expression vector for beta-galactosidase. It was used as a control vector and designated “None” in the figures. It was also used to adjust total DNA content in transfection experiments.

### Cell culture, lentiviral vector production and generation of stable populations

293T (ATCC CRL-3216), HeLa (ATCC CCL-2) and 208F (ECACC 85103116) cells were maintained at 37°C, 8% CO2, in DMEM with 10% heat-inactivated FCS, 100u/mL penicillin and 100μg/mL streptomycin (PS). MCF10A cells (ATCC CRL-10317) were cultured at 37°C, 10% CO2, in DMEM:F12 medium supplemented with 5% horse serum, 5ng/mL EGF (Peprotech), 10μg/mL insulin (Sigma), 1ng/mL cholera toxin (Sigma), 100μg/mL hydrocortisone (Sigma) and PS. Unless specified all reagents were from Life Technology. Lentiviral particles were produced as described [43] using JetPrime (PolyPlus Transfection). Cells were infected with viral supernatants and selected with Hygromycin B (46u/mL, Calbiochem) 3 days later. The populations of resistant cells were thereafter maintained in selection media.

### Signalling experiments

Cells in 12-well plates were transfected with 250ng total DNA (50 or 30ng of Env in 293T and HeLa cells respectively, supplemented with pCMV-ß) using 1.25μL of Fugene6 (Promega). Media were replaced 18 hours post transfection (without FCS for Hela cells to minimize background). When used, inhibitors were added during the medium change (FTI-277 (5μM, Sigma), TAK-632 (5μM, Selleckchem), U0126 (5μM, Cell Signaling)). TNFα was used at 100ng/μL (R&D Systems). Cells were used for protein or RNA extraction 48 hours post transfection. For Western blot analysis, cells were lysed in PBS, 1% NP40 or RIPA (Life Technology) complemented with Halt protease and phosphatase inhibitor cocktail (ThermoScientific). Cell lysates were then subjected to SDS-PAGE as described [43]. Proteins of interest were detected using antibodies from Cell Signalling: p44/42 MAPK, phospho-p44/42 MAPK (Thr202/Tyr204), p38 MAPK, phospho-p38 MAPK (Thr180/Tyr182), pan-Akt, phospho-Akt, IKBα or Sigma (Tubulin). HERV-K(HML2) anti-Env antibody was previously described [61]. MLV ampho Env protein was detected using a goat antiserum directed against Rauscher leukemia virus gp70 (from the National Cancer Institute, Frederick, MD). HERV-K Rec protein was detected using a polyclonal rabbit antiserum given by R. Löwer. HRP-conjugated secondary antibodies (GE Healthcare Or Dako) and ECL Plus Reagent (GE Healthcare) were used for Western blots. Membrane stripping was done using ReBlot Plus Strong (Merck Millipore).

### RNA isolation, qRT-PCR, gene expression profiling

Total RNA were extracted with the RNeasy extraction kit (Qiagen) and treated with DNase I (Ambion). For microarray experiments, we compared duplicates of RNA from 293T transfected with either HERV-K(HML2) Env or the control plasmid, HERV-K(HML2) Env-LP, collected 24 and 48 hours post-transfection. Gene expression analysis was performed on Agilent SurePrint G3 Human GE 8x60K Microarrays (Agilent Technologies, AMADID 39494). Data were extracted using Feature Extraction software (v10.5.1.1; Agilent Technologies) and normalized using an empirical Bayes method. Top-ranked genes were selected for an absolute fold-change ≥2 using a False Detection Rate (FDR) <0.05.

DNase-treated RNAs were reverse-transcribed using the MLV reverse-transcriptase (Applied Biosystems). qPCR was performed using the QuantiFast SYBR Green PCR kit (Qiagen) on the ABI PRISM 7000 system. Efficacy of the PCR reaction was checked for each primer pair. Transcript levels were normalized to RPLO employing the ΔΔCt method.

### Transwell migration and invasion assays

MCF10A cells were resuspended in DMEM:F12 media without serum or additional additives. 5x10^4^ cells in 500μL were seeded into the top of each transwell (Corning, 24-well inserts, 8μm pore), and 750μL of complete culture medium was added to the bottom of each well as a chemoattractant.

The cells were incubated for 22 hours before non-migratory cells were removed and the membrane fixed with methanol. Membranes were stained with DAPI and the migrated cells counted. Invasion assays were performed similarly except transwells were precoated with 16μg of Matrigel (Corning).

### Quantification of cellular morphological changes

MCF10A cell populations were incubated in DMEM/F12 media supplemented with 5μM CellTracker Green (Invitrogen) for 30 minutes, and then cultured for an additional 60 minutes in complete media before fixation in 4% PFA. Cells were imaged to assess changes in morphology. Average cell size (area) was calculated by measuring the surface area covered by the cells (green stain) relative to the number of cells.

### Transformation assays

208F cells (seeded at 2x10^5^ cells per 3.5 cm dish the day before) were transfected with 4μg DNA using Fugene 6 (Promega). After 24h, they were reseeded in a 10 cm dish. When the cells reached confluence, medium was replaced by DMEM complemented with PS, 5% FCS and 1μM dexamethasone. Medium was replaced weekly. After 3–4 weeks, cells were stained with Leishmann to allow counting of transformed foci.

## Supporting information

S1 Fig The expression level of HERV-K Env transcripts in stably transduced MCF10A cells was assessed by qRT-PCR and compared to the parental control as well as other cell lines including some derived from germ cell tumors (NCCIT, 2102Ep) and melanomas (SKMel28, WM3526, WM3682). (PDF)

S2 Fig Clustal alignment of the HERV-K Env endogenous alleles compared to the consensus protein. The leader peptide region is highlighted in grey, the furin cleavage site is in red and the predicted transmembrane region is highlighted in yellow.
(PDF)

S3 Fig Clustal alignment of betaretroviral Env proteins. The sequences of endogenous HERV-K, JSRV (consensus and enJSRV-18 resp.) as well as infectious MMTV and JSRV Envs are compared. Leader peptides are highlighted in grey, the furin cleavage sites are in red and the predicted transmembrane regions are highlighted in yellow. The PI3K/Akt binding motif (YXXM), only present in infectious JSRV and necessary for transformation [7, 68], is highlighted in green.
(PDF)
