# Supplementary figures and images for: A human endogenous retrovirus-derived gene that can contribute to oncogenesis by activating the ERK pathway and inducing migration and invasion
Source: PLoS Pathog. 2017 Jun 26;13(6):e1006451. doi: 10.1371/journal.ppat.1006451 (PMC5501692; doi:10.1371/journal.ppat.1006451)

Supporting Figure 1

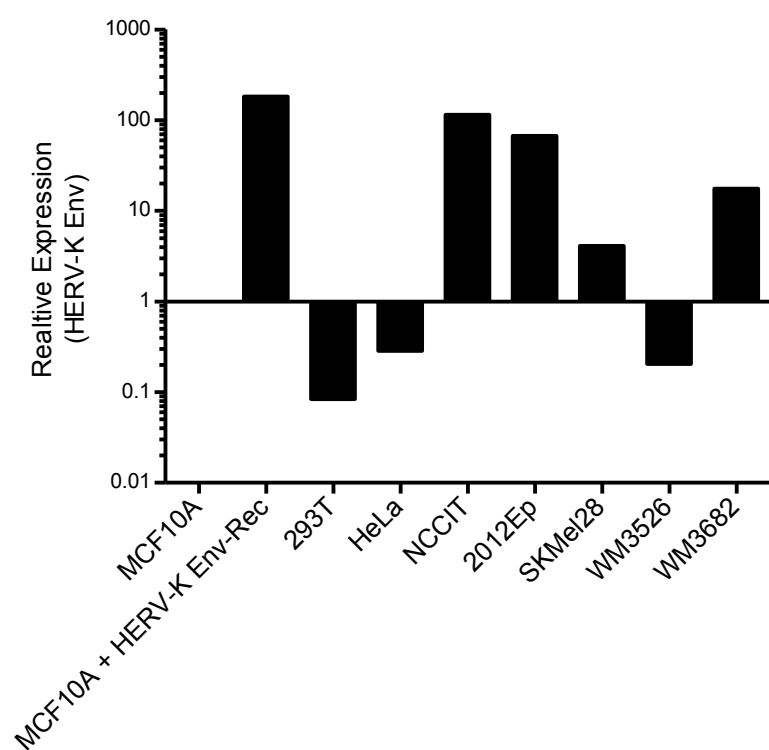

Supplement: S1 Fig — (PDF) [file ppat.1006451.s001.pdf]
