# Supplementary material for: A human endogenous retrovirus-derived gene that can contribute to oncogenesis by activating the ERK pathway and inducing migration and invasion
Source: PLoS Pathog. 2017 Jun 26;13(6):e1006451. doi: 10.1371/journal.ppat.1006451 (PMC5501692; doi:10.1371/journal.ppat.1006451)
Supplement: S2 Fig — The leader peptide region is highlighted in grey, the furin cleavage site is in red and the predicted transmembrane region is highlighted in yellow. (PDF) [file ppat.1006451.s002.pdf]

Supporting Figure 2:

```
                  10        20        30        40        50        60        70        80        90       100       110       120       130       140       150
K-cons    MNPSEMQRKAPPRRRRHRNRAPLTHKMNKMVTSEEQMKLPSTKKAEPPTWAQLKKLTQLATKYLENTKVTQTPESMLLAALMIVSMVVSLPMPAGAAAANYTYWAYVPFPPLIRAVTWMDNPIEVYVNDSVWVPGPIDDRCPAKPEEEGM
K-108     ..........................................................................................................................T...........................
K-109     ......................................................................................................N...............................................
K-113     ............................................................................................................................I...........T..C..........
K-115     .................................................................................................V....N...............................................
K-17833   ........................................................................................................................................T..H..........
K-74261   .H............................-.......................................................................N..............................H................

                 160       170       180       190       200       210       220       230       240       250       260       270       280       290       300
K-cons    MINISIGYRYPPICLGRAPGCLMPAVQNWLVEVPTVSPISRFTYHMVSGMSLRPRVNYLQDFSYQRSLKFRPKGKPCPKEIPKESKNTEVLVWEECVANSAVILQNNEFGTIIDWAPRGQFYHNCSGQTQSCPSAQVSPAVDSDLTESLD
K-108     ........H..............................C..............................................................................................................
K-109     ...........-......................I....C...........................................................................T.Q................................
K-113     ...............................................................................................................L......................................
K-115     ......................................................................................................................................................
K-17833   ...................................................................F..................................................................................
K-74261   ........H...................................N.......................................................V.................................................

                 310       320       330       340       350       360       370       380       390       400       410       420       430       440       450
K-cons    KHKHKKLQSFYPWEWGEKGISTPRPKIISPVSGPEHPELWRLTVASHHIRIWSGNQTLETRDRKPFYTVDLNSSLTVPLQSCVKPPYMLVVGNIVIKPDSQTITCENCRLLTCIDSTFNWQHRILLVRAREGVWIPVSMDRPWEASPSIH
K-108     ...........................V........................................I...............................................................................V.
K-109     ............................................................................L.........................................................................
K-113     ......................A.............................................I...............................................................................V.
K-115     ..................R........V................................................L.......................................................................V.
K-17833   ..........................................................................V.......I.............................................................T.....
K-74261   ......................................................................................................................................................

                 460       470       480       490       500       510       520       530       540       550       560       570       580       590       600
K-cons    ILTEVLKGVLNRSKRFIFTLIAVIMGLIAVTATAAVAGVALHSSVQSVNFVNDWQKNSTRLWNSQSSIDQKLANQINDLRQTVIWMGDRLMSLEHRFQLQCDWNTSDFCITPQIYNESEHHWDMVRRHLQGREDNLTLDISKLKEQIFEA
K-108     ......................................................................................................................................................
K-109     .....................................................G................................................................................................
K-113     .......................................................N......................................................................C.......................
K-115     .....................................................G..............................................................D.................................
K-17833   T...........................................................................................................S.........................................
K-74261   ................................M..........F..........................................................................................................

                 610       620       630       640       650       660       670       680       690
K-cons    SKAHLNLVPGTEAIAGVADGLANLNPVTWVKTIGSTTIINLILILVCLFCLLLVCRCTQQLRRDSDHRERAMMTMAVLSKRKGGNVGKSKRDQIVTVSV   699
K-108     ...................................................................................................   699
K-109     ...................................................................................................   698
K-113     .........................T............................Y....................V.......................   699
K-115     ...................................................................................................   699
K-17833   ...................................................................................................   699
K-74261   ........................................................F........Y.........V.......................   698
```
