# Supplementary material for: A human endogenous retrovirus-derived gene that can contribute to oncogenesis by activating the ERK pathway and inducing migration and invasion
Source: PLoS Pathog. 2017 Jun 26;13(6):e1006451. doi: 10.1371/journal.ppat.1006451 (PMC5501692; doi:10.1371/journal.ppat.1006451)
Supplement: S3 Fig — The sequences of endogenous HERV-K, JSRV (consensus and enJSRV-18 resp.) as well as infectious MMTV and JSRV Envs are compared. Leader peptides are highlighted in grey, the furin cleavage sites are in red and the predicted transmembrane regions are highlighted in yellow. The PI3K/Akt binding motif (YXXM), only present in infectious JSRV and necessary for transformation [7, 68], is highlighted in green. (PDF) [file ppat.1006451.s003.pdf]

Supporting Figure 3:

```
HERV-K      -----------------MNPSEMQRKAPPRRRRHRNRAPLTHKMNKMVTSEEQMKLPSTKK
MMTV        MPKHQSGSPIGSSDLLLSGKKQRPHLALRRKRRREMRKINRKVRRMNLAPI---------
JSRV        MPKRRAGFRKGWYA-------------------RQRNSLTHQMQRMTLSEP--------T
enJSRV18    MPKRRAGFRKGWYA-------------------RQRNSLTHQMHRMTLSEP--------T
                                             *:   :.:::.:*  :

HERV-K      AEPPTWAQLKKLTQLATKYLENTKVTQTPESMLLAALMIVSMVVSLPMPAGAAAANYTYW
MMTV        KEKTAWQHLQALIF-E-A-EEVLKTSQTPQTSLTLFLALLSV---LG---PPPVTGESYW
JSRV        SELPTQRQIEALMRYAWN-EAHVQPPVTPTNILIMLLLL--L---QR---VQNGAAAAFW
enJSRV18    SELPTQRQIEALMRYAWN-EAHVQPPVTPTKILIMLLLL--L---QR---IQNGAASAFW
             *  :  ::: *           :   ** . *   * :  :            :  ::*

HERV-K      AYVPFPPLIRAVTWMDN-PIEVYVNDSVWVPGPIDDRCPAKPE----EEGMMINISIGYR
MMTV        AYLPKPPILHPVGWGNTDPIRVLTNQTIYLGGSPDFHGFRNMSGNVHFEGKSD-------
JSRV        AYIPDPPMIQSLGWDRE-IVPVYVNDTSLLGGKSDIHIS-PQQANISFYGLTT-------
enJSRV18    AYIPDPPMIQSLGWDKE-TVPVYVNDTSLLGGKSDIHIS-PQQANISFYGLTT-------
            **:* **::: : *     : * .*::  : *  * :     .      *

HERV-K      YPPICLG----RAPGCLMPAVQNWLVEVPTVSPISRFTYHMVSGMSLRPRV--NYLQDFS
MMTV        TLPICFSFSFSTPTGCFQVDKQVFLSDTPTVDNNKP------GGKGDKRRMWELWLTTLG
JSRV        QYPMCFSYQSQHP-HCIQVSADISYPRVTISGIDEK------TGKK-----------SYG
enJSRV18    QYPMCFSYQSQHP-HCIQVSADISYPRVTISGIDEK------TGKR-----------SYR
              *:*:.        *:    :     .   .  .        *

HERV-K      YQ-RSLKFRPKGKPCPKEIPKES-----------KNTEVLVWEECVANSAV--ILQNNEF
MMTV        NSGANTKLVPIKKKLPPKYPHCQIAFKKDAFWEGDESAPPRWLPCAFPDQGVSFSPKGAL
JSRV        NGS-----------GPLDIPFCDKHLSI------GIGIDTPWTLCRARVASVYNINNANA
enJSRV18    DGT-----------GPLDIPFCDKNLSI------GIGIDTPWTLCRARIASVYNINNANT
                           * . *  .                  *  *           :

HERV-K      GTIIDWAPRGQFYHNCSGQTQSCPSAQVSPAVDSDLTESLDKHKHKKLQSFYP-------
MMTV        GLLWDFSLPSPSVDQSDQIKSK-------KDLFGNYTPPV----NKEVHRWYEAGWVEPT
JSRV        TFLWDWAPGGTP-DFPEYRGQH-------PPIFSVNTAP---------------------
enJSRV18    TLLWDWAPGGTP-DFPEYRGQH-------PPILSVNTAP---------------------
              : *::   .   .  .   .          : .  *

HERV-K      WEWGEKGISTPRPKIISPVSGPEHPELWRLTVASHHIRIWSGNQTLETRDRKPFYTVDLN
MMTV        WFWENSPKD---PNDRDFTALVPHTELFRLVAA----------SRYLILKR-----PGFQ
JSRV        ---------------------IYQTELWKLLAAFGHGN-----SLYLQPNISGSKYGDVG
enJSRV18    ---------------------IFQTELWKLLAAFGHGN-----SLYLQPNISGSKYGDVG
                                   : **::* .*          .     .       ..

HERV-K      SSLTVPLQSCVKPPYMLVVGN-----IVIKPDSQTITCENCRLLTCIDSTFNWQHRILLV
MMTV        EHDMIPTSACVTYPHAILLGLPQLIDIEKRGSTFHISCSSCRLTNCLDSSAYDYA-AIIV
JSRV        VTGFLYPRACVPYPFMLIQGH---MEITLSLNIYHLNCSNCILTNCIRGVAKGEQ-VIIV
enJSRV18    VTGFLYPRACVPYPFMLIQGH---MEITLSLNIYHLNCSNCILTNCIRGVAKGEQ-VIIV
                :   :**  *. :: *      *    .   :.*..* * .*: .        ::*

HERV-K      RAREGVWIPVSMD-RPWEASPSIHILTEVLKGVLNRSKRFIFTLIAVIMGLIAVTATAAV
MMTV        KRPPYVLLPVDIGDEPWFDDSAIQTF-R-YATDLIRAKRFVAAIILGISALIAIITSFAV
JSRV        KQPAFVMLPVEIA-EAWYDETALELLQR-INTALSRPKRGLSLIILGIVSLITLIATAVT
enJSRV18    KQPAFVMLPVEIT-EEWYDETALELLQR-INTALSRPKRGLSLIILGIVSLITLIATAVT
            :    *  :**.:  .  *  .  ::. :  .     *  *  ** :  :*  *  .**:: ::  ..

HERV-K      AGVALHSSVQSVNFVNDWQKNSTRLWNSQSSIDQKLANQINDLRQTVIWMGDRLMSLEHR
MMTV        ATTALVKEMQTATFVNNLHRNVTLALSEQRIIDLKLEARLNALEEVVLELGQDVANLKTR
JSRV        ASVSLAQSIQAAHTVDSLSYNVTKVMGTQEDIDKKIEDRLSALYDVVRVLGEQVQSINFR
enJSRV18    ASVSLAQSIQAAHTVDSLSYNVTKVMGTQEDIDKKIEDRLSALYDVVRVLGEQVQSINFR
            * .:* ..:*:.  *:.   *  *   .  *  ** *:  ::. *  :.*  :*:  : .::  *
```

```
HERV-K        FQLQCDWNTSDFCITPQIYNESEHHWDMVRRHLQGR--EDNLTLDISKLKEQIFEASKAH
MMTV          MSTRCHANYDFICVTPLPYNASE-SWERTKAHLLGIWNDNEISYNIQELTNLISDMSKQH
JSRV          MKIQCHANYKWICVTKKPYNTSDFPWDKVKKHLQGIWFNTNLSLDLLQLHNEILDIENSP
enJSRV18      MKIQCHANYKWICVTKKPYNTSDFPWDKVKKHLQGIWFNTNVSLDLLQLHNEILDIENSP
              :. :*. * . :*:*   ** *:  *: .: ** *   : ::: :: :* : * : .:

HERV-K        LNLVPGTEAIAGVADGLANLNPVTWVKT---IGSTTIINLILILVCLFCLLLVCRCTQQL
MMTV          IDTVDLSGLAQSFANGVKALNPLDWTQYFIFIGVGALLLV--IVLMIFPIVFQCLAKSLD
JSRV          KATLNIADTVDNFLQNLFSNFPSLHSLWKTLIGVGILVFI--IIV--VILIFPCLVRGMV
enJSRV18      KATLNIADTVDNFLQNLFSNFPSLHSLWRSIIAMGAVLTV--VLI--IICLAPCLIRSIV
                 :  :    .. :.:    *         *.   :: :  :::  .  :  *

HERV-K        RRDSDHRE-----------RAMMTMAVLSKRKGGNVGKSKRDQIV-TVSV-
MMTV          QVQSDLN----------------VLLLKKKKGGNAAPAAEMVELPRVSYT
JSRV          RDFLKMRVEMLHMKYRNMLQHQHLMELLKNKERGDAGDDP-----------
enJSRV18      KEFLHMRVLI----HKNMLQHQHLMELLKNKERGAAGDDP-----------
              :   . .                 : :*.::: * ..
```
